# Association of Proton Pump Inhibitor Use with Cancer in Patients Undergoing Maintenance Hemodialysis: A Population-Based Cohort Study

**DOI:** 10.3390/jcm15030920

**Published:** 2026-01-23

**Authors:** Seok Hui Kang, So Young Park, Yu Jeong Lim, Bo Yeon Kim, Ji Young Choi, Jun-Young Do

**Affiliations:** 1 Division of Nephrology, Department of Internal Medicine, College of Medicine, Yeungnam University, Daegu 42415, Republic of Korea; kangkang@ynu.ac.kr; 2 Department of Physiology, College of Medicine, Yeungnam University, Daegu 42415, Republic of Korea; 3 Health Insurance Review and Assessment Service, Wonju 26465, Republic of Korea

**Keywords:** cancer, proton pump inhibitor, hemodialysis, mortality

## Abstract

**Background**: Despite widespread proton pump inhibitor (PPI) use in hemodialysis (HD), evidence on cancer risk in high-risk populations remains scarce. We investigated the association between PPI use and incident cancer in a population-based cohort of patients receiving HD. **Methods**: We used data from the 4th–7th HD quality assessments from South Korea and data linked to claims and death. We classified patients by PPI prescription over 1 year, including No-Prescription (no PPI during the year, *n* = 37,934); Short (PPI for <60 days, *n* = 9909); and Long (PPI for ≥60 days, *n* = 18,108) groups. Any cancer-free survival and overall survival by PPI use were evaluated. **Results**: The 5-year cancer-free rates for any cancer were 89.6%, 88.5%, and 88.1% in the No-Prescription, Short, and Long groups, respectively. The 5-year patient survival rate was 42.2%, 43.8%, and 40.3% in the No-Prescription, Short, and Long groups, respectively. Patients prescribed PPI had a higher cancer risk than those without a PPI prescription. However, survival among patients with cancer did not differ significantly across the three groups. The Long group had a higher risk of pancreatic and renal cancers than the other two groups. The No-Prescription group had lower risks of thyroid, prostate, and liver cancers than the other groups. **Conclusions**: In our study, long-term PPI use was associated with higher overall cancer risk, particularly pancreatic and renal cancers, compared with the No-Prescription group. Although PPI prescription did not significantly affect cancer-specific survival, the findings suggest that prolonged PPI use may contribute to cancer development in this population.

## 1. Introduction

Chronic kidney disease is increasing worldwide because of population aging and the rising prevalence of metabolic and cardiovascular conditions. It poses a major public health challenge, driving substantial healthcare expenditure and often progressing to end-stage kidney disease requiring dialysis or transplantation. Among available modalities, hemodialysis (HD) remains the most widely used treatment for end-stage kidney disease. Although patients receiving HD have markedly lower survival than the general population, advances in dialysis technology, improved medical care, and earlier correction of modifiable risks have gradually improved survival [1]. As survival improves, non–dialysis-related complications have become major clinical concerns. HD patients have a higher malignancy risk than the general population, likely due to uremic toxin accumulation, immune dysfunction, chronic inflammation, and a high comorbidity burden [2]. However, studies that delineate cancer risk factors specifically in HD remain limited.

Proton pump inhibitors (PPIs) are among the most frequently prescribed drugs worldwide. By suppressing gastric acid secretion, they are widely used to treat peptic ulcer disease, gastritis, and gastroesophageal reflux. In HD, gastrointestinal symptoms are common because of uremia, cytokine imbalance, and polypharmacy, prompting frequent PPI use. Emerging evidence suggests a possible association between long-term PPI use and increased cancer risk. Population-based studies have reported links between PPI prescription and gastric, pancreatic, and colorectal cancers [3,4,5,6,7,8,9], although findings are conflicting [8,10]. Despite widespread PPI use in HD, evidence on cancer risk in high-risk population remains scarce. We therefore investigated the association between PPI use and incident cancer in a population-based cohort of patients receiving HD.

## 2. Materials and Methods

### 2.1. Data Sources and Study Population

In the Republic of Korea, national HD quality assessment programs are conducted for quality assurance [11]. This study utilized nationwide data collected during four rounds of the HD quality assessment program—namely, the fourth–seventh cycles conducted between 2013 and 2021. Eligible participants were adults aged 18 years or older who received maintenance HD for a minimum of three months and were treated at least twice weekly. We retrospectively analyzed Health Insurance Review and Assessment Service (HIRA) assessment data linked to claims and death. Of 127,302 participants, across the fourth–seventh assessments, only the first record for individuals with multiple sentries (*n* = 53,728) was retained. We excluded those undergoing HD with a catheter (*n* = 1763); those with insufficient data (*n* = 271); and those with International Classification of Diseases, 10th Revision (ICD-10) cancer codes during the 1-year window spanning the 6 months before and after the index assessment (*n* = 5589). After applying these criteria, the study included 65,951 patients. The research protocol was approved by the Institutional Review Board of a tertiary medical center (approval number: YUMC 2023-12-012; approval date: 15 December 2023). Informed consent was waived because records were anonymized and de-identified before the analysis.

### 2.2. PPI Prescription

PPI prescriptions were identified using medication codes (Table S1). We classified patients by PPI prescription during the 1-year window spanning the 6 months before and after the index HD quality assessment, creating No-Prescription (no PPI during the year); Short (PPI for <60 days); and Long (PPI for ≥60 days) groups. In our cohort, patients with cancer codes identified during the 1-year window spanning 6 months before and 6 months after the index assessment were excluded from the analysis (*n* = 5589). Prior to the application of this exclusion criterion, the proportions of patients with cancer were 2824 (6.9%) in the No-Prescription group, 904 (8.4%) in the Short group, and 1861 (9.3%) in the Long group, respectively. All analyses were conducted after excluding patients with any cancer codes in the preceding 1-year period. Given that the proportion of patients with pre-existing cancer was below 10% in each prescription group, it is unlikely that the exclusion of these patients materially influenced the overall direction or robustness of the study findings.

### 2.3. Study Variables

We extracted demographic and dialysis-related information, including sex, duration of HD (in months), age, type of vascular access, and primary cause of end-stage kidney disease. Data collected at each assessment encompassed body mass index (kg/m^2^); dialysis adequacy measured by Kt/V_urea_; ultrafiltration volume per session (L/session); hemoglobin concentration (g/dL); serum albumin (g/dL); and serum levels of phosphorus, creatinine, and calcium (mg/dL). These variables were recorded on a monthly basis, and laboratory measurements were summarized as mean values over the observation period. Dialysis adequacy (Kt/V_urea_) was estimated using a previous formula [12].

Concomitant medications—renin–angiotensin system blockers (RASBs), aspirin, clopidogrel, statins, and histamine H_2_-receptor blockers—were identified by medication codes; use was defined as ≥1 prescription during the assessment period. Before HD quality assessments were conducted, comorbidities were evaluated for 1 year and defined using the Charlson comorbidity index (CCI), which encompasses 17 different comorbid conditions [13]. CCI scores were derived at the individual patient level, while myocardial infarction (MI) and congestive heart failure (CHF) were identified using corresponding ICD-10 codes.

The use of immunosuppressive agents or kidney transplantation may influence the outcome. In our study, immunosuppressive agents such as steroids (prednisolone, methylprednisolone, deflazacort)—as well as antimetabolite (mycophenolate mofetil, mycophenolic acid, and azathioprine), cyclophosphamide, and calcineurin inhibitors (tacrolimus and cyclosporine)—were evaluated. We evaluated the use of direct oral anticoagulants (DOACs), including apixaban, dabigatran, rivaroxaban, and edoxaban. Patients with a history of kidney transplantation were identified using ICD-10 codes Z94.0 and T86.1 in the preceding year.

### 2.4. Outcomes

All participants were followed until June 2024. Mortality data were obtained from the HIRA database. Patients who transitioned from maintenance HD to peritoneal dialysis or underwent kidney transplantation before any study outcome were censored at the date of modality change or transplantation. Incident cancer was identified by newly recorded ICD-10 codes for the 12 most prevalent cancers in South Korea, as reported in the 2020 national cancer statistics [14]. The included cancer types were stomach (C16), colorectal (C18–21), liver (C22), gallbladder or biliary duct (C23–C24), pancreas (C25), lung (C33–C34), breast (C50), uterus or cervix (C53–55), prostate (C61), kidney (C64–C65), bladder (C67), and thyroid (C73).

The start of follow-up for cancer incidence analysis was defined as the end date of each corresponding HD quality assessment cycle. The date of cancer diagnosis was defined as the first date on which an ICD-10 code for any of the specified cancers appeared in the HIRA claims database after this start date. The cancer-free interval was calculated from the end of the relevant HD quality assessment cycle (time zero) to the earliest occurrence of one of the following events: (1) newly identified cancer diagnosis, (2) death, or (3) censoring at the end of follow-up. For patients who developed cancer during follow-up, survival analysis was performed with time zero defined as the date of cancer diagnosis. Cancer-specific survival time was calculated from the date of cancer diagnosis to death or censoring at the last available follow-up.

### 2.5. Statistical Analyses

All statistical analyses were conducted using SAS Enterprise Guide v.7.1 (SAS Institute Inc., Cary, NA, USA) and R v.3.5.1 (R Foundation for Statistical Computing, Vienna, Austria). Descriptive statistics are presented as counts with percentages for categorical variables and as means with standard deviations for continuous variables. Group comparisons for categorical variables were performed using the chi-square test or Fisher’s exact test, as appropriate. Continuous variables were compared using one-way analysis of variance, with Tukey’s test applied for post hoc multiple comparisons.

Time-to-event outcomes were evaluated using the Kaplan–Meier approach, and survival curves were compared across groups using the log-rank test. Associations between exposure and outcomes were quantified by hazard ratios (HRs) with corresponding confidence intervals derived from Cox proportional hazards models. Multivariable Cox regression analyses included adjustment for demographic factors (age and sex), body mass index, dialysis-related variables (vascular access type, dialysis vintage, ultrafiltration volume, and Kt/V_urea_), comorbidity burden (CCI score, diabetes, MI, and CHF), laboratory parameters (hemoglobin, albumin, creatinine, calcium, and phosphorus), and concomitant medication use (RASBs, aspirin, clopidogrel, statins, and H_2_-receptor antagonists). All covariates were entered into the models simultaneously. Prespecified subgroup analyses were performed according to sex, age category, and dichotomized CCI score. To explore potential non-linear associations between the duration of PPI prescription and cancer incidence, restricted cubic spline functions were applied. A *p*-value below 0.05 was considered statistically significant.

We performed additional sensitivity analyses using a propensity score-weighted cohort. We used propensity score weighting to balance these characteristics and ensure that the results of our analyses were not biased. We created the balanced cohort for the three groups using generalized boosted models for the following variables: age; sex; body mass index; the underlying cause of end-stage kidney disease; types of vascular access; CCI score; HD vintage; ultrafiltration volume; Kt/V_urea_; hemoglobin, creatinine, phosphorus, and calcium levels; the use of aspirin, statins, RASB, clopidogrel, or H2-receptor blockers; and the presence of MI or CHF. Propensity scores were used to calculate the inverse probability of treatment weights. Finally, we defined the balanced cohort as the weighted sample, and continuous variables were presented as means and standard errors. *p*-values were tested using a general linear model with a complex survey design, including sample weights.

## 3. Results

### 3.1. Baseline Patient Characteristics

The number of patients in the No-Prescription group was 37,934, while the Short and Long groups contained 9909 and 18,108 patients, respectively (Table 1).

The distribution of underlying causes of end-stage kidney disease differed across groups. In the No-Prescription group, the number of patients with diabetes, hypertension, glomerulonephritis, other causes, and unknown causes was 16,693 (44.0%), 10,205 (26.9%), 3969 (10.5%), 3034 (8.0%), and 4033 (10.6%), respectively. The corresponding figures for in the Short group were 4471 (45.1%), 2593 (26.2%), 956 (9.6%), 817 (8.2%), and 1072 (10.8%), and those for the Long group were 9103 (50.3%), 4393 (24.3%), 1530 (8.4%), 1336 (7.4%), and 1746 (9.6%). The most frequently prescribed PPIs in the Short group were esomeprazole (2783 patients, 28.1%), rabeprazole (2276, 23.0%), pantoprazole (1888, 19.1%), lansoprazole (1734, 17.5%), omeprazole (751, 7.6%), dexlansoprazole (342, 3.5%), and ilaprazole (135, 1.4%). In the Long group, rabeprazole (5015, 27.7%) was the most commonly prescribed PPI, followed by esomeprazole (4078, 22.5%), pantoprazole (3726, 20.6%), lansoprazole (3230, 17.8%), omeprazole (1368, 7.6%), dexlansoprazole (421, 2.3%), and ilaprazole (270, 1.5%). Overall, esomeprazole and rabeprazole were the most commonly prescribed PPIs in the both groups.

The proportions of female sex, arteriovenous graft, and MI or CHF were lower in the No-Prescription group than in the other groups. HD vintage was longer in the No-Prescription group than in the other groups. The CCI scores were highest in the Long group among the three groups. Levels of hemoglobin, albumin, phosphorus, calcium, or creatinine were lower in the Long group than in the other two groups. The proportions of diabetes and the use of RASBs, aspirin, clopidogrel, statins, or H_2_-receptor blockers were higher in the Long group than in the other groups.

We evaluated the number of HD sessions per week. The proportion of patients undergoing two HD sessions per week was 1417 (3.7%) in the No-Prescription group, 340 (3.4%) in the Short group, and 599 (3.3%) in the Long group. Although the proportion of patients receiving twice-weekly HD was slightly higher in the No-Prescription group than in the other groups, the vast majority of patients across all groups underwent thrice-weekly HD. Therefore, differences in dialysis schedule are unlikely to have materially influenced the overall results.

The number of patients receiving DOACs was 93 (0.2%) in the No-Prescription group, 42 (0.4%) in the Short group, and 112 (0.6%) in the Long group, respectively. Because the proportion of patients receiving DOACs was very small across all groups, it is unlikely that DOAC use had a substantial impact on the overall study outcomes. In addition, the limited number of DOAC prescriptions precluded meaningful subgroup or stratified analyses.

### 3.2. Cancer Risk and Patient Survival According to PPI Prescription

Follow-up duration differed across groups: 59 ± 34 months in the No-Prescription group, 66 ± 36 months in the Short group, and 63 ± 35 months in the Long group. At the end of follow-up, the outcomes in the No-Prescription group were 18,296 (48.2%) survivors, 16,369 (43.2%) deaths, 132 (0.2%) transfers to peritoneal dialysis, and 3137 (8.3%) kidney transplantations; in the Short-group, the outcomes were 4878 (49.2%), 4114 (41.5%), 30 (0.3%), and 887 (9.0%); and in the Long group, the outcomes were 8460 (46.7%), 8489 (46.9%), 70 (0.4%), and 1089 (6.0%), respectively (*p* < 0.001).

**Figure 1:**
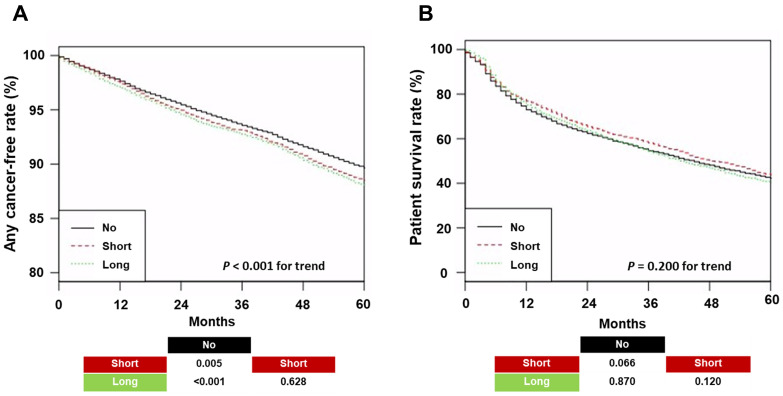
Kaplan–Meier curves for any cancer-free survival and overall survival by proton pump inhibitor use. (**A**) Any cancer-free survival in the total cohort; (**B**) overall survival among patients with cancer. Pairwise log-rank *p*-values are shown at the bottom of the graph. Abbreviations: No, patients without a prescription for proton pump inhibitor use during a year; Short, patients with prescriptions for <60 days during a year; Long, patients with prescriptions for ≥60 days during a year.

The 5-year cancer-free rates for any cancer were 89.6%, 88.5%, and 88.1% in the No-Prescription, Short, and Long groups, respectively (Figure 1A).

The 5-year patient survival rate in patients with cancer was 42.2%, 43.8%, and 40.3% in the No-Prescription, Short, and Long groups, respectively (Figure 1B). Patients prescribed PPI had a higher cancer risk than those without a PPI prescription. However, survival among patients with cancer did not differ significantly across the three groups.

**Figure 2:**
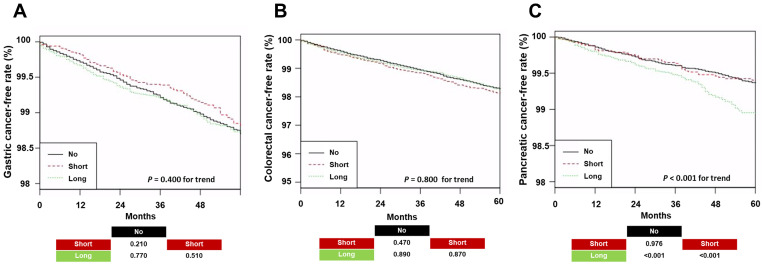
Kaplan–Meier curves for cancer-free survival by proton pump inhibitor use: (**A**) gastric; (**B**) colorectal; (**C**) pancreatic. Pairwise log-rank *p*-values are shown at the bottom of the graph. Abbreviations: No, patients without a prescription for proton pump inhibitor use during a year; Short, patients with prescriptions for <60 days during a year; Long, patients with prescriptions for ≥60 days during a year.

There were no significant differences in gastric or colorectal cancer incidence among the three groups, but the Long group had a higher risk of pancreatic cancer than the other two groups (Figure 2).

The No-Prescription group had lower risks of thyroid, prostate, and liver cancers than the other groups (Figure S1).

### 3.3. Cox Regression and Subgroup Analyses

Multivariable Cox regression indicated that the No-Prescription group had a lower risk of any cancer than the other two groups (Table 2).

However, the Long group had better patient survival than the No-Prescription group. The Long group had higher HRs for pancreatic and renal cancers than the other two groups (Table S2). The No-Prescription group had lower HRs for thyroid and liver cancers than the other two groups. Subgroup analyses showed that findings in participants younger than 65 years and in men were similar to those in the total cohort (Table 3).

Among participants with low CCI scores, those in the Long group had a higher HR than those in the No-Prescription group. In addition, we plotted a spline curve based on the association between prescription days and incident cancer risk, which revealed a possible dose-dependent relationship (Figure S2).

Overall, kidney cancer was the most common cancer among all patients and in the No-Prescription group, whereas liver cancer was the most frequently observed cancer in the Short and Long groups (Table S3). The number (proportions) of patients who received kidney transplantation in the No-Prescription group was 1058 (2.8%), while there were 297 (3.0%) in the Short group and 506 (2.8%) in the Long group. Given that the proportion was below 3% in each prescription group, these patients are unlikely to have materially influenced the overall direction or robustness of the study findings. Almost all renal cancers are likely to develop in the native kidney.

The number (proportions) of patients who received immunosuppressive agents in the No-Prescription group was 5966 (15.7%), while there were 2057 (20.8%) in the Short group and 3694 (20.4%) in the Long group. In addition, we performed subgroup analyses based on the use of immunosuppressive agents (Table S4). Although the overall statistical significance was attenuated compared with the total cohort analysis, the trend toward higher HRs with PPI prescription was maintained, and no significant interaction by immunosuppressive agent use was observed (*p* = 0.556 and *p* = 0.541 in univariable and multivariable analyses of cancer incidence; *p* = 0.337 and *p* = 0.564 in univariable and multivariable analyses of patient survival).

### 3.4. Analyses Using Weighted Cohort

Balance among the three groups was assessed by calculating the maximum pairwise absolute standardized mean differences in covariates before and after weighting (Figure S3). After applying weights, the maximum absolute standardized mean differences and differences in baseline characteristics decreased for most covariates. The number of patients in the No-Prescription, Short, and Long groups in the weighted cohort was 65,446, 64,085, and 64,043, respectively. Baseline characteristics after weighting are shown in Table S5, and differences in most characteristics were attenuated after weighting. In the weighted cohort, the cancer-free rates were 89.4%, 88.7%, and 88.2% in the No-Prescription, Short, and Long groups, respectively (Figure S4A). The survival rates among patients with cancer were 41.2%, 42.2%, and 44.3% in the No-Prescription, Short, and Long groups, respectively (Figure S4B). The Long group had the highest incidence of cancer and the highest survival among patients with cancer among the three groups. Furthermore, univariable and multivariable Cox regression analyses revealed that the Short or Long group had a higher risk of cancer than the No-Prescription group (Table S6). Among patients with cancer, those prescribed a PPI had a lower risk of mortality than those without a PPI prescription. Results from the weighted cohort showed a similar trend to those from the cohort without weighting.

## 4. Discussion

We included 65,951 patients undergoing maintenance HD without a recent cancer diagnosis and evaluated the association between PPI use and incident cancer. The 5-year cancer-free rate was lower in the Long group than in the No-Prescription group, and multivariable Cox regression analyses showed similar trends. Subgroups with low CCI scores, men, and younger participants showed patterns consistent with the overall cohort. Among patients diagnosed with cancer, survival did not differ significantly across the three groups. Pancreatic cancer risk was highest in the Long group, and thyroid and liver cancer risks were lower in the No-Prescription group than in the other groups.

PPIs, introduced in the 1980s, are widely used to treat peptic ulcer disease, gastroesophageal reflux disease, and gastritis [15]. Reports have suggested that long-term PPI use may increase cancer risk [3,4,5,6,7,8,9,16]. Although mechanisms remain incompletely defined, studies indicate that PPI use disrupts the gut microbiome, potentially increasing colorectal cancer risk [17]. Other studies have reported that PPIs elevate gastrin, promoting gastric epithelial proliferation and gastric cancer risk [18]. Because dialysis patients already exhibit dysbiosis and relatively elevated gastrin compared with the general population, PPI use in HD may further increase cancer risk through additive effects [19,20].

Epidemiological studies of PPI use and cancer risk have yielded inconsistent results. Several population-based studies reported higher risks of gastric, pancreatic, or colorectal cancer with long-term PPI use [3,5,6,7,8,9,21], whereas others found no significant association [8,10,22,23]. In contrast, our study evaluated a large homogeneous cohort receiving maintenance HD, whose metabolic and immunologic milieu differs from that of the general population. The chronic uremic milieu, oxidative stress, and dysbiosis characteristic of HD may amplify the oncogenic potential of PPI-induced changes. Furthermore, PPI use in HD is often prolonged and at higher doses because of persistent gastrointestinal symptoms and polypharmacy, increasing cumulative prescription and risk. These pathophysiological and clinical differences may partly explain the more consistent association between PPI use and increased cancer incidence observed in our study.

Notably, the magnitude of risk varied by cancer type. Pancreatic, prostatic, and renal cancers showed stronger associations with long-term PPI use, whereas thyroid and liver cancers were less frequently observed in the No-Prescription group. The potential increase in pancreatic cancer risk has been hypothesized to be related to hypergastrinemia-induced pancreatic cell proliferation following sustained acid suppression; however, this mechanism remains speculative and has not been conclusively established [24]. Similarly, PPI-associated dysbiosis and bacterial translocation may contribute to systemic inflammation, which has been proposed as a pathway involved in pancreatic and renal carcinogenesis, although direct evidence in dialysis populations is limited [25,26]. Conversely, the lower incidence of thyroid and liver cancers among the No-prescription group may reflect confounding by indication—patients with advanced comorbidities or gastrointestinal symptoms requiring PPIs often undergo more surveillance and imaging, increasing detection of some cancers. Alternatively, hepatic metabolism and altered drug clearance in HD could modulate organ-specific susceptibility. Mechanistic and longitudinal studies are needed to clarify these differential associations and to determine whether specific cancer subtypes are particularly sensitive to PPI-related biology in dialysis. Taken together, the cancer type-specific associations observed in this study should be interpreted in the context of incidence rather than prognosis, as no corresponding adverse impact on post-diagnosis survival was identified.

An important finding of this study is that, although long-term PPI use was associated with a higher incidence of cancer, PPI prescription was not associated with poorer cancer-specific survival. Moreover, adjusted overall survival among patients with prolonged PPI use was not inferior and was even numerically better in some analyses. These findings suggest that the observed association between PPI use and cancer incidence may reflect increased detection, surveillance bias, or shared underlying risk factors rather than more aggressive cancer biology or adverse cancer prognosis. The lack of an association between PPI use and cancer-specific survival further supports the interpretation that PPI prescription is unlikely to be linked to tumor aggressiveness or progression; alternatively, more frequent clinical encounters and earlier cancer detection among long-term PPI users may have contributed to more favorable overall survival.

Importantly, the statistically significant associations observed in this study should be clearly distinguished from clinical relevance. Although long-term PPI use was associated with an increased incidence of cancer, the magnitude of this association was modest, and no corresponding adverse effect on cancer-specific survival was identified. From a clinical perspective, these findings suggest that the observed associations, while statistically detectable in a large population-based cohort, may not necessarily translate into meaningful differences in individual patient prognosis or cancer-related outcomes. Therefore, the results should be interpreted as indicative of epidemiological association rather than evidence of clinically actionable harm, particularly in the absence of a demonstrated impact on post-diagnosis survival.

Considering the inherently elevated risk of renal cancer in patients with end-stage kidney disease and the higher proportion of liver cancer observed in patients with PPI prescription, these findings may suggest a potential association between PPI prescription and liver cancer. However, more robust epidemiological evidence and mechanistic studies are required to clarify cancer type-specific risks and underlying biological pathways.

Subgroup findings warrant comment. Stronger associations in the low CCI, male, and younger subgroups likely reflect fewer competing comorbidities, allowing the effect of PPI use to emerge more clearly. The stronger signal in men may also reflect a higher baseline cancer risk. These patterns require confirmation and elucidation of underlying mechanisms.

Our dataset did not include data on residual renal function. The HD quality assessment program was designed to focus primarily on patient management and treatment performance at the HD center level rather than on detailed patient-level physiological parameters. To facilitate the construction of a nationwide population-based database, variables that were difficult to measure in a standardized manner were not systematically collected. Although residual renal function is an important prognostic factor in patients undergoing HD, it was not included in the assessment because urine collection, measurement techniques, and calculation methods can vary substantially across centers, and reliable assessment is also influenced by patient compliance. Nevertheless, patients in our dataset had a relatively long dialysis vintage, with a mean duration of approximately 49–50 months. Among patients with a dialysis duration exceeding four years, clinically meaningful residual renal function is uncommon; therefore, it is unlikely that the omission of this variable had a substantial impact on the observed clinical outcomes.

The 60-day cut-off was selected a priori as a pragmatic threshold within a 1-year observation window to distinguish patients with intermittent or short-term PPI exposure from those with more sustained use. This cut-off has been commonly applied in claims-based pharmaco-epidemiologic studies to define meaningful exposure while maintaining adequate group sizes and statistical stability. Importantly, we acknowledge that this threshold is not intended to represent a precise biological boundary but rather an operational definition based on prescription duration. To address this limitation, we emphasize that the 60-day cut-off was chosen pragmatically and that residual exposure misclassification is possible. We also wish to clarify that our findings should be interpreted in the context of prescription duration rather than cumulative dose and that future studies incorporating dose, intensity, or time-varying exposure metrics are warranted.

Although data on the total number of prescribed days of PPI use were available in this study, information on prescribed dose, actual intake dose, and medication adherence was not available. In addition, some patients may have used multiple PPIs sequentially or in combination, and claims data cannot verify whether prescribed medications were actually taken as directed. Therefore, dose-specific effects and PPI type-specific risks could not be reliably assessed in this retrospective claims-based study. Such analyses would require prospective studies with direct assessment of medication intake or post hoc analyses of randomized controlled trials that include detailed adherence data.

This study has limitations. First, it is a retrospective analysis of prevalent patients undergoing HD. Therefore, causal inference is limited, and selection bias is possible given the baseline imbalances between groups. We addressed these concerns with multivariable adjustment and subgroup analyses. Second, cancer incidence was <20% at 5 years, and it was low overall. Limited events may reduce statistical power, making small or nonspecific PPI effects difficult to detect. Event counts may also have been too small to observe statistical significance. Third, cancer ascertainment relied solely on diagnostic codes; stage and pathology data were unavailable. These limitations may have affected incidence estimates and survival analyses. Nevertheless, cancer coding is relatively accurate. In South Korea, assignment of a cancer code confers substantial tax, medical, and insurance benefits and is restricted to cases with clear pathologic or radiologic evidence; the HIRA also audits entries. Accordingly, the accuracy of ICD-10 cancer codes in Korea is considered high. Fourth, laboratory findings were not standardized in our study. Biochemical data included serum calcium, phosphorus, albumin, creatinine, and hemoglobin, all of which were entered as measured values through a standardized web-based reporting system. Although the units of measurement for each parameter were uniform across centers, specific assay methods were not strictly mandated, except for hemoglobin, for which spectrophotometric measurement is recommended in the HD quality assessment program. Nevertheless, in routine clinical practice in Korea, serum albumin is most commonly measured using the bromocresol green method, and serum creatinine is predominantly assessed using the kinetic Jaffe method [27]. Therefore, while inter-laboratory variability in assay methods cannot be excluded, the biochemical measurements in this study are considered to reflect routine clinical practice in Korean laboratories. Fifth, in this study, detailed information on HD modality and membrane type was not available. In South Korea, reimbursement for HD is provided under a bundled payment system rather than a fee-for-service model. Under this system, a fixed reimbursement is paid by patients and insurers for each HD session, and individual centers have discretion in selecting dialysis modalities and membrane types within this reimbursement framework. Consequently, claims data related to HD capture only the occurrence and frequency of dialysis sessions under a single procedure code, without providing detailed information on modality or membrane characteristics. In addition, the HD quality assessment program does not include data on dialysis modality. Despite these data limitations, it is reasonable to assume that, under a fixed reimbursement structure, most centers prioritize conventional HD from a cost-efficiency perspective. Supporting this interpretation, national registry data from Korea indicate that fewer than 12% of patients receive hemodiafiltration at least once per week, suggesting that conventional HD remains the predominant modality in routine clinical practice [28].

Sixth, information on prior peritoneal dialysis before HD initiation could be informative for understanding modality transitions and total dialysis exposure. However, in the present dataset, the evaluable claims history was limited to 6 months prior to each HD quality assessment cycle. Dialysis vintage was therefore defined based on the duration of maintenance HD before the start of the HD quality assessment program, rather than on total renal replacement therapy duration across modalities. Consequently, we were unable to reliably identify patients who had received peritoneal dialysis before HD initiation or to account for prior peritoneal dialysis exposure in the analysis. This limitation has been acknowledged, as the lack of complete pre-HD modality history may result in some underestimation of total dialysis duration. Nevertheless, national registry data from Korea indicate that peritoneal dialysis accounts for less than 5% of all renal replacement therapy modalities [28]. Therefore, the proportion of patients who transitioned from peritoneal dialysis to HD is expected to be relatively small, and the absence of information on prior peritoneal dialysis exposure is unlikely to have materially influenced the overall direction or robustness of our findings. Future studies incorporating longer longitudinal claims data or registry-based modality histories would be required to more comprehensively evaluate the impact of prior PD exposure on clinical outcomes.

In this large, nationwide cohort of HD patients, long-term PPI use was associated with a higher overall incidence of cancer, particularly pancreatic and renal cancers, compared with the No-Prescription group. However, PPI prescription was not significantly associated with cancer-specific survival, suggesting that this association may be related to cancer occurrence rather than disease progression or prognosis. These findings represent observational associations and should be interpreted cautiously. Further studies are needed to clarify whether the observed association reflects residual confounding, surveillance effects, or underlying biological mechanisms. Nevertheless, careful consideration of the clinical indications and duration of PPI therapy may be warranted in HD patients.

## Figures and Tables

**Table 1:** Baseline characteristics.

	No-Prescription (*n* = 37,934)	Short Group (*n* = 9909)	Long Group (*n* = 18,108)	*p*-Value
Age (years)	59.9 ± 13.4	60.1 ± 13.2	62.5 ± 12.4 ^a,b^	<0.001
Male sex	23,492 (61.9%)	5596 (56.5%)	10,450 (57.7%)	<0.001
Body mass index (kg/m^2^)	22.7 ± 3.5	22.7 ± 3.6	22.7 ± 3.5	0.799
Hemodialysis vintage (months)	54 ± 59	50 ± 58 ^a^	49 ± 57 ^a^	<0.001
Diabetes as underlying cause of ESKD	16,693 (44.0%)	4471 (45.1%)	9103 (50.3%)	<0.001
CCI score	6.9 ± 2.9	7.7 ± 2.8 ^a^	8.5 ± 2.8 ^a,b^	<0.001
Arteriovenous fistula	32,711 (86.2%)	8360 (84.4%)	15,206 (84.0%)	<0.001
Kt/V_urea_	1.52 ± 0.25	1.53 ± 0.25	1.53 ± 0.25	0.148
Ultrafiltration volume (L/session)	2.32 ± 0.96	2.31 ± 0.92	2.26 ± 0.93 ^a,b^	<0.001
Hemoglobin (g/dL)	10.7 ± 0.8	10.7 ± 0.8	10.6 ± 0.8 ^a,b^	<0.001
Serum albumin (g/dL)	4.01 ± 0.33	4.00 ± 0.33 ^a^	3.96 ± 0.34 ^a,b^	<0.001
Serum phosphorus (mg/dL)	5.04 ± 1.29	5.06 ± 1.30	4.74 ± 1.24 ^a,b^	<0.001
Serum calcium (mg/dL)	8.89 ± 0.78	8.88 ± 0.76	8.82 ± 0.75 ^a,b^	<0.001
Serum creatinine (mg/dL)	9.62 ± 2.77	9.41 ± 2.70 ^a^	9.12 ± 2.69 ^a,b^	<0.001
RASB use	23,802 (62.7%)	6748 (68.1%)	12,551 (69.3%)	<0.001
Aspirin use	8637 (22.8%)	2686 (27.1%)	5918 (32.7%)	<0.001
Clopidogrel use	4465 (11.8%)	1532 (15.5%)	4082 (22.5%)	<0.001
Statin use	15,836 (41.7%)	4762 (48.1%)	9744 (53.8%)	<0.001
H_2_-receptor blocker use	15,916 (42.0%)	4807 (48.5%)	10,196 (56.3%)	<0.001
MI or CHF	11,912 (31.4%)	4043 (40.8%)	6412 (35.4%)	<0.001

Data are presented as mean ± SD for continuous variables and numbers (percentage) for categorical variables. Group comparisons were carried out using one-way analysis of variance with Tukey post hoc tests; categorical variables were tested via Pearson’s chi-square tests. Abbreviations: CCI, Charlson Comorbidity Index; CHF, congestive heart failure; ESKD, end-stage kidney disease; MI, myocardial infarction; RASB, renin–angiotensin system blocker; No-Prescription, patients without proton pump inhibitor prescription for 1 year; Short, patients with prescription for <60 days in 1 year; Long, patients with prescription for ≥60 days in 1 year. ^a^ *p* < 0.05 vs. No-Prescription; ^b^ *p* < 0.05 vs. Short group.

**Table 2:** Proton pump inhibitor prescription and any cancer or mortality.

	Univariable		Multivariable	
	HR (95% CI)	*p*-Value	HR (95% CI)	*p*-Value
Any cancer				
Ref: No-Prescription				
Short	1.10 (1.03–1.18)	0.004	1.09 (1.02–1.16)	0.014
Long	1.12 (1.06–1.18)	<0.001	1.07 (1.01–1.13)	0.025
Ref: Short				
Long	1.02 (0.95–1.10)	0.610	0.98 (0.91–1.05)	0.601
Mortality				
Ref: No-Prescription				
Short	0.92 (0.84–1.01)	0.063	0.92 (0.84–1.01)	0.090
Long	0.99 (0.93–1.07)	0.875	0.92 (0.85–0.99)	0.025
Ref: Short				
Long	1.08 (0.98–1.20)	0.116	1.00 (0.90–1.10)	0.927

Multivariable models adjusted for age, sex, vascular access type, hemodialysis vintage, underlying cause of end-stage kidney disease; Charlson Comorbidity Index score; Kt/V_urea_; ultrafiltration volume; hemoglobin; serum albumin, creatinine, phosphorus, and calcium; use of renin–angiotensin system blocker, statins, clopidogrel, aspirin, or H_2_-receptor blockers; and presence of myocardial infarction or congestive heart failure; models were fitted using the enter method. Abbreviations: HR, hazard ratio; CI, confidence interval; No-Prescription, patients without proton pump inhibitor prescription for 1 year; Short, patients with prescription for <60 days in 1 year; Long, patients with prescription for ≥60 days in 1 year.

**Table 3:** Prescription of proton pump inhibitor and any cancer risk by subgroups.

	Univariable		Multivariable			Univariable		Multivariable	
	HR (95% CI)	*p*	HR (95% CI)	*p*		HR (95% CI)	*p*	HR (95% CI)	*p*
<65 years					≥65 years				
Ref: No-Prescription									
Short	1.14 (1.04–1.24)	0.003	1.11 (1.02–1.21)	0.019		1.05 (0.95–1.17)	0.311	1.05 (0.95–1.17)	0.325
Long	1.14 (1.06–1.23)	<0.001	1.10 (1.02–1.19)	0.013		1.04 (0.96–1.12)	0.362	1.03 (0.95–1.12)	0.457
Ref: Short									
Long	1.00 (0.91–1.10)	0.967	0.99 (0.90–1.09)	0.878		0.98 (0.88–1.10)	0.777	0.98 (0.88–1.09)	0.715
Male					Female				
Ref: No-Prescription									
Short	1.14 (1.05–1.24)	0.002	1.10 (1.01–1.20)	0.027		1.08 (0.97–1.20)	0.144	1.06 (0.96–1.18)	0.266
Long	1.16 (1.09–1.24)	<0.001	1.07 (1.00–1.15)	0.042		1.09 (0.99–1.19)	0.067	1.06 (0.97–1.17)	0.178
Ref: Short									
Long	1.02 (0.93–1.12)	0.648	0.98 (0.89–1.07)	0.627		1.00 (0.89–1.13)	0.947	1.00 (0.89–1.13)	0.966
CCI < 7					CCI ≥ 7				
Ref: No-Prescription									
Short	0.98 (0.88–1.09)	0.730	0.98 (0.88–1.10)	0.764		1.15 (1.06–1.25)	0.001	1.15 (1.06–1.25)	<0.001
Long	1.16 (1.06–1.27)	0.001	1.13 (1.03–1.24)	0.010		1.06 (0.99–1.14)	0.070	1.04 (0.97–1.12)	0.229
Ref: Short									
Long	1.18 (1.04–1.35)	0.010	1.15 (1.01–1.31)	0.033		0.93 (0.85–1.01)	0.094	0.90 (0.83–0.99)	0.024

Multivariable models adjusted for age, sex, vascular access type, hemodialysis vintage, underlying cause of end-stage kidney disease; Charlson Comorbidity Index score; Kt/V_urea_; ultrafiltration volume; hemoglobin; serum albumin, creatinine, phosphorus, and calcium; use of renin–angiotensin system blockers, statins, clopidogrel, aspirin, or H_2_-receptor blockers; and presence of myocardial infarction or congestive heart failure; models were fitted using the enter method. Abbreviations: HR, hazard ratio; CI, confidence interval; No-Prescription, patients without proton pump inhibitor prescription for 1 year; Short, patients with prescription for <60 days in 1 year; Long, patients with prescription for ≥60 days in 1 year.

## Data Availability

Raw data were generated at the Health Insurance Review and Assessment Service. The database can be requested from the Health Insurance Review and Assessment Service by sending a study proposal, including the purpose of the study, study design, and duration of analysis, via the following website: https://www.hira.or.kr. The authors cannot distribute the data without permission.

## Supplementary Materials

The following supporting information can be downloaded at: https://www.mdpi.com/article/10.3390/jcm15030920/s1, Table S1. Medication types and Health Insurance Review and Assessment Service codes; Table S2. Proton pump inhibitor prescription and site-specific cancer risk; Table S3. Cancer types among patients diagnosed with cancer; Table S4. Prescription of proton pump inhibitors and any cancer risk, stratified by immunosuppressive agent use; Table S5. Baseline characteristics after weighting; Table S6. Proton pump inhibitor prescription and any cancer or mortality in the weighted cohort; Figure S1. Kaplan–Meier curves of cancer-free rates by proton pump inhibitor use; Figure S2. Spline curve plotting hazard ratio and 95% confidence interval for cancer incidence according to prescription days of proton pump inhibitors; Figure S3. Balance test; Figure S4. Kaplan–Meier curves for any cancer-free survival and overall survival by proton pump inhibitor use.
