# Supplementary material for: Association of Proton Pump Inhibitor Use with Cancer in Patients Undergoing Maintenance Hemodialysis: A Population-Based Cohort Study
Source: J Clin Med. 2026 Jan 23;15(3):920. doi: 10.3390/jcm15030920 (PMC12898568; doi:10.3390/jcm15030920)
Supplement: Supplementary file 1 [file jcm-15-00920-s001.zip › jcm-4074630-supplementary.pdf]

Table S1. Medication types and Health Insurance Review and Assessment Service codes.

Medications	Codes
**Alpril**	104201, 104202
**Benazepril**	114701
**Captopril**	122901, 122902, 122903
**Cilazapril**	133001, 133002, 133003
**Enalapril**	151601, 151603
**Fosinopril**	163501, 163502
**Imidapril**	173401, 173402
**Moexipril**	196801, 196802
**Lisinopril**	184501
**Perindopril**	211301, 211302, 501601, 501602
**Quinapril**	221901,
**Ramipril**	222401, 222402, 222404
**Zofenopril**	510401, 510402, 510403
**Temocapril**	235002
**Delapril**	140901, 140902
**Captopril + hydrochlorothiazide**	262200, 262300
**Enalapril + hydrochlorothiazide**	440300, 453700, 453600
**Ramipril + felodipine**	447100, 447200
**Ramipril + hydrochlorothiazide**	448600, 448700
**Perindopril + indapamide**	556200
**Lisinopril + hydrochlorothiazide**	499200, 499300
**Moexipril + hydrochlorothiazide**	440800, 497900
**Enalapril + nitrendipine**	466000
**Candesartan**	122601, 122602, 122603
**Irbesartan**	177301, 177303
**Losartan**	185701, 185702
**Valsartan**	247101, 247102, 247103, 247104

**Fimasartan**	515201, 515202, 515203
**Azilsartan**	662401, 662402, 662403
**Telmisartan**	378801, 378802
**Eprosartan**	429201
**Olmesartan**	468501, 468502, 468503, 520901, 520902
**Valsartan + amlodipine**	492800, 492900, 495800, 522600, 522700, 522800, 522900, 523000, 523100, 523200, 523300, 523400
**Valsartan + lercanidipne**	522200. 522300. 522400
**Valsartan + pitavastatin**	634900, 635000, 635100, 635200
**Valsartan + sacubitril**	651401, 651402, 651403
**Valsartan + rosuvastatin**	629700, 629800, 525000, 525100, 525200, 525300,
**Valsartan + hydrochlorothiazide**	356400, 442600
**Olmesaetan + amlodipine**	500500, 500600, 547500. 547600, 547700, 547800, 547900, 548000, 582200, 582400, 629400, 629500, 629600, 631300, 632800, 632900, 633000
**Olmesartan + hydrochlorothiazide**	513600
**Olmesartan + hydrochlorothiazide + amlodipine**	519700, 519800, 519900, 520000, 520100
**Olmesartan + rosuvastatin**	653200, 644100, 644200, 526300, 526400, 526500, 526900
**Telmisartan + hydrochlorothiazide**	502600, 443200, 443300
**Telmisartan + rosuvastatin**	629900, 630000, 630100, 630200, 631600, 631700
**Telmisartan + amlodipine**	511500, 511600, 511700, 521200, 521300, 521400, 623100, 644800
**Telmisartan+ hydrochlorothiazide + amlodipine**	663500, 663600, 663700, 663800
**Telmisartan + rosuvastatin + amlodipine**	671700, 671600, 671500, 671400, 671300, 671200,
**Losartan + hydrochlorothiazide**	262500, 378900, 486900
**Losartan + amlodipine**	502700, 503000, 513900, 637400, 637500, 637600
**Losartan+ rosuvastatin+ amlodipine**	663900, 664000, 664100, 664200, 664300, 664400,
**Losartan+chlorthalidone+ amlodipine**	662800, 662900, 663000
**Fimasartan + hydrochlorothiazide**	522000, 526800

**Fimasartan + amlodipine**	651900, 652000, 652100, 652700, 651900
**Fimasartan + rosuvastatin**	654600, 654700, 654800, 654900, 655000
**Candesartan + hydrochlorothiazide**	423700
**Candesartan + amlodipine**	652900, 653000, 653100, 652900, 652900
**Candesartan + rosuvastatin**	673700, 661800, 661900, 662000, 662100
**Irbesartan + hydrochlorothiazide**	385700, 385800
**Irbesartan + atorvastatin**	527000, 527100, 524000, 524100
**Azilsartan + chlorthalidone**	673500, 673600
**Eprosartan + hydrochlorothiazide**	460500
**Amlodipine**	495901, 459802, 483201, 486501, 107601, 107601, 459801, 459801, 459901, 464601, 470801, 476201, 479701, 483202, 486502, 107602, 107602, 470802
**Amlodipine + atorvastatin**	614500, 472300, 472400, 472500, 518900
**Amlodipine + rosuvastatin**	673900, 674000, 674100
**Amosulalol**	107901, 107902
**Arotinolol**	110202, 110201
**Nolol**	483102, 111402, 483101, 111403, 111401
**Nolol + chlorthalidone**	262100, 460200
**Barnidipine**	114003, 114001, 114002
**Benidipine**	115101, 115102, 115104, 115103
**Betaxolol**	116801, 116803
**Bevantolol**	117002, 117001
**Bisoprolol**	117904, 117903, 117902, 117901
**Bisoprolol + hydrochlorothiazide**	469800, 470000, 469900
**Carteolol**	124801
**Carvedilol**	125005, 125003, 662201, 125008, 125001, 662202, 125007, 125002, 125006, 125004
**Celiprolol**	129101
**Cilnidipine**	133102, 133101

**Clonidine**	136505
**Diltiazem**	145706, 145707, 145707, 145703, 145706, 145707
**Doxazocin**	149101, 149102, 149104, 149103
**Efonidipine**	441202, 441201
**Felodipine**	157503, 157501
**Felodipine + metoprolol**	262400
**Hydralazine**	170701
**Lacidipine**	180301, 180302, 180303
**Lercanidipine**	182001, 182002
**Manidipine**	188001, 188002
**Metoprolol**	194003, 193802, 262400
**Metoprolol + hydrochlorothiazide**	262600
**Metoprolol + felodipine**	262400
**Minoxidil**	196102
**Nadolol**	198301
**Nebivolol**	489501, 489502, 489503
**Nicardipine**	201003, 201002
**Nifedipine**	201407, 201405, 528201, 201409, 528202, 201401, 201401, 201408
**Nimodipine**	201901, 356202, 356203, 356201, 356202
**Nisoldipine**	356202
**Propranolol**	219901, 219904, 219906, 219905
**Terazosin**	235501, 235502, 235503, 616501
**Verapamil**	247606, 247607, 247603, 247605, 247601
**Atorvastatin + ezetimibe**	633800, 633900, 634800
**Pitavastatin + fenofibr**	679300
**Rosuvastatin + ezetimibe**	640700, 640800, 640900

**Aspirin**	110701, 110702, 110801, 110802, 111001, 111001, 111001, 111002, 111003, 111003
**Clopidogrel**	133201, 133201, 133201, 133202, 133203, 506100
**Cilostazol**	136901, 492501, 495201, 498801, 501501
**Ticlopidine**	498900, 239201, 239202
**Aspirin + bethocarbamol**	256800
**Aspirin + clopidogrel**	517900, 517900, 517900, 667500
**Aspirin + dipyridamole**	489700
**Atorvastatin**	111502, 502202, 633900, 472400, 518900, 524100, 527000, 672000, 672100, 111503, 502203, 634800, 472500, 111504, 502204
**Fluvastatin**	162401, 162402, 162403
**Lovastatin**	185801
**Pitavastatin**	470901, 470902, 470903
**Pravastatin**	216601, 216602, 216603, 216604
**Rosuvastatin**	454001, 454002, 454002, 454003, 454003, 454005
**Simvastatin**	227801, 227802, 227803, 227805, 227806
**Dexlansoprazole**	621901, 621902
**Esomeprazole**	509901, 367202, 498002, 367202, 509902, 518000, 367201, 523500, 498001, 527400, 367201, 670700, 459401
**Ilaprazole**	505501
**Lansoprazole**	181301, 181301, 181301, 181302, 181302, 181302, 181302
**Omeprazole**	204403, 204401, 204401, 664500, 204402, 640200, 204501
**Pantoprazole**	519202, 519202, 208901, 519201, 208802, 519201, 208902, 208801, 519203, 656701, 208801
**Cimetidine**	133301, 133302, 133303, 133305, 133301, 133330
**Famotidine**	157301, 157302, 157303, 157302
**Nizatidine**	202701, 202704
**Ranitidine**	222801, 222803, 222805, 222830, 222831, 222802, 222804
**Roxatidine**	225201, 225202

**Ranitidine + sucralfate + tripotassium bismuth**	271800, 631800
**Lafutidine**	489302

Table S2. Proton pump inhibitor prescription and site-specific cancer risk.

	Univariable		Multivariable	
	**HR (95% CI)**	***P***	**HR (95% CI)**	***P***
**Stomach cancer**				
Ref: No-Prescription				
Short	0.88 (0.71–1.08)	0.203	0.88 (0.71–1.08)	0.213
Long	0.98 (0.83–1.15)	0.788	0.88 (0.75–1.04)	0.132
Ref: Short				
Long	1.12 (0.89–1.41)	0.341	1.00 (0.80–1.27)	0.969
**Colorectal cancer**				
Ref: No-Prescription				
Short	1.06 (0.90–1.25)	0.465	1.07 (0.91–1.26)	0.436
Long	1.01 (0.88–1.16)	0.899	0.94 (0.82–1.08)	0.372
Ref: Short				
Long	0.95 (0.79–1.14)	0.579	0.88 (0.73–1.06)	0.171
**Pancreatic cancer**				
Ref: No-Prescription				
Short	1.00 (0.76–1.30)	0.978	0.90 (0.69–1.18)	0.450
Long	1.63 (1.36–1.97)	<0.001	1.35 (1.11–1.64)	0.003
Ref: Short				
Long	1.64 (1.24–2.17)	<0.001	1.49 (1.13–1.98)	0.005
**Bladder cancer**				
Ref: No-Prescription				
Short	1.12 (0.85–1.48)	0.409	1.13 (0.86–1.49)	0.388
Long	1.10 (0.87–1.38)	0.433	0.99 (0.78–1.26)	0.951
Ref: Short				
Long	0.98 (0.72–1.33)	0.873	0.88 (0.64–1.20)	0.410
**Thyroid cancer**				
Ref: No-Prescription				
Short	1.39 (1.14–1.70)	0.001	1.34 (1.09–1.64)	0.005
Long	1.24 (1.05–1.48)	0.014	1.35 (1.12–1.61)	0.001
Ref: Short				
Long	0.90 (0.72–1.12)	0.333	1.01 (0.80–1.26)	0.946
**Lung cancer**				
Ref: No-Prescription				
Short	1.10 (0.92–1.31)	0.291	1.09 (0.91–1.30)	0.347
Long	1.16 (1.01–1.34)	0.034	1.04 (0.90–1.21)	0.570
Ref: Short				
Long	1.10 (0.87–1.28)	0.556	0.96 (0.79–1.16)	0.667
**Breast cancer**				
Ref: No-Prescription				
Short	0.98 (0.74–1.29)	0.865	1.01 (0.76–1.34)	0.943
Long	1.03 (0.82–1.30)	0.775	1.15 (0.91–1.46)	0.236
Ref: Short				
Long	1.06 (0.78–1.44)	0.716	1.14 (0.84–1.56)	0.403
**Prostate cancer**				
Ref: No-Prescription				
Short	1.51 (1.23–1.85)	<0.001	1.40 (1.14–1.73)	0.001
Long	1.20 (0.99–1.44)	0.053	1.03 (0.86–1.25)	0.737
Ref: Short				
Long	0.80 (0.63–1.00)	0.054	0.74 (0.58–0.93)	0.011
**Liver cancer**				
Ref: No-Prescription				
Short	1.25 (1.07–1.48)	0.006	1.20 (1.02–1.42)	0.026

Long	1.36 (1.19–1.54)	<0.001	1.22 (1.06–1.40)	0.004
Ref: Short				
Long	1.08 (0.91–1.29)	0.387	1.01 (0.85–1.21)	0.895
**Gallbladder and biliary duct cancer**				
Ref: No-Prescription				
Short	1.16 (0.82–1.62)	0.407	1.13 (0.80–1.59)	0.497
Long	1.12 (0.84–1.48)	0.435	1.04 (0.78–1.40)	0.775
Ref: Short				
Long	0.97 (0.66–1.41)	0.870	0.93 (0.63–1.36)	0.695
**Renal cancer**				
Ref: No-Prescription				
Short	0.95 (0.80–1.13)	0.554	0.98 (0.83–1.17)	0.857
Long	1.11 (0.98–1.27)	0.106	1.21 (1.06–1.39)	0.006
Ref: Short				
Long	1.17 (0.97–1.41)	0.095	1.23 (1.02–1.48)	0.031
**Uterus or cervical cancer**				
Ref: No-Prescription				
Short	1.24 (0.88–1.74)	0.222	1.07 (0.75–1.51)	0.722
Long	0.87 (0.64–1.20)	0.411	0.84 (0.60–1.17)	0.293
Ref: Short				
Long	0.71 (0.47–1.06)	0.093	0.77 (0.52–1.18)	0.248

Multivariable models adjusted for age, sex, vascular access type, hemodialysis vintage, underlying cause of end-stage kidney disease; Charlson Comorbidity Index score; Kt/V_{urea}; ultrafiltration volume; hemoglobin; serum albumin, creatinine, phosphorus, and calcium; use of renin-angiotensin system blockers, statins, clopidogrel, aspirin, or H_2-receptor blockers; and presence of myocardial infarction or congestive heart failure; models were fitted using the enter method.

Abbreviations: HR, hazard ratio; CI, confidence interval; No-Prescription, patients without proton pump inhibitor prescription for 1 year; Short, patients with prescription for < 60 days in 1 year; Long, patients with prescription for ≥ 60 days in 1 year.

Table S3. Cancer types among patients diagnosed with cancer

	Total (n = 62,951)	No-Prescription (n = 37,934)	Short (n = 9,909)	Long (n = 18,108)
**Thyroid**	688 (1.0%)	369 (1.0%)	127 (1.3%)	192 (1.1%)
**Lung**	1039 (1.6%)	584 (1.5%)	161 (1.6%)	294 (1.6%)
**Colorectum**	1183 (1.8%)	695 (1.8%)	185 (1.9%)	303 (1.7%)
**Stomach**	829 (1.3%)	504 (1.3%)	111 (1.1%)	214 (1.2%)
**Breast**	427 (0.6%)	243 (0.6%)	67 (0.7%)	117 (0.6%)
**Prostate**	648 (1.0%)	356 (0.9%)	121 (1.2%)	171 (0.9%)
**Liver**	1160 (1.8%)	610 (1.6%)	192 (1.9%)	358 (2.0%)
**Pancreas**	524 (0.8%)	270 (0.7%)	67 (0.7%)	187 (1.0%)
**Gall bladder or bile duct**	263 (0.4%)	148 (0.4%)	43 (0.4%)	72 (0.4%)
**Kidney**	1199 (1.8%)	703 (1.9%)	166 (1.7%)	330 (1.8%)
**Uterus or cervix**	234 (0.4%)	139 (0.4%)	43 (0.4%)	52 (0.3%)
**Bladder**	405 (0.6%)	230 (0.6%)	65 (0.7%)	110 (0.6%)

Proportions were calculated using each group total as the denominator. Some patients were diagnosed with more than one primary cancer during follow-up.

Abbreviations: No-Prescription, patients without a proton pump inhibitor prescription within 1 year; Short, patients with a prescription for < 60 days within 1 year; Long, patients with a prescription for ≥ 60 days within 1 year.

Table S4. Prescription of proton pump inhibitors and any-cancer risk, stratified by immunosuppressive agent use

	Univariable		Multivariable	
	HR (95% CI)	*P*	HR (95% CI)	*P*
**ISA (+)**				
**Any-cancer**				
Ref: No-Prescription				
Short	1.04 (0.91–1.20)	0.547	1.01 (0.88–1.17)	0.867
Long	1.15 (1.02–1.29)	0.019	1.07 (0.95–1.21)	0.257
Ref: Short				
Long	1.10 (0.94–1.28)	0.221	1.06 (0.91–1.23)	0.465
**Mortality**				
Ref: No-Prescription				
Short	1.01 (0.83–1.22)	0.920	1.02 (0.84–1.24)	0.865
Long	0.96 (0.82–1.12)	0.588	0.95 (0.81–1.12)	0.541
Ref: Short				
Long	0.95 (0.77–1.17)	0.613	0.93 (0.76–1.15)	0.527
**ISA (-)**				
**Any-cancer**				
Ref: No-Prescription				
Short	1.11 (1.03–1.20)	0.006	1.10 (1.02–1.18)	0.014
Long	1.11 (1.04–1.18)	0.001	1.05 (0.99–1.12)	0.127
Ref: Short				
Long	1.00 (0.92–1.08)	0.939	0.96 (0.88–1.04)	0.296
**Mortality**				
Ref: No-Prescription				
Short	0.89 (0.80–0.99)	0.032	0.91 (0.82–1.02)	0.094
Long	1.01 (0.93–1.09)	0.824	0.92 (0.84–0.99)	0.040
Ref: Short				
Long	1.13 (1.01–1.27)	0.037	1.00 (0.89–1.13)	0.967

Multivariable models were adjusted for age, sex, vascular access type, hemodialysis vintage, underlying cause of end-stage kidney disease, Charlson Comorbidity Index score, Kt/V_{urea}; ultrafiltration volume, hemoglobin, serum albumin, creatinine, phosphorus, and calcium, use of renin-angiotensin system blockers, statins, clopidogrel, aspirin, or H_2-receptor blockers, and presence of myocardial infarction or congestive heart failure. Models were fitted using the enter method.

Abbreviations: HR, hazard ratio; CI, confidence interval; ISA, immunosuppressive agents; No-Prescription, patients without a proton pump inhibitor prestcription within 1 year; Short, patients with a prescription for < 60 days within 1 year; Long, patients with a prescription for ≥ 60 days within 1 year.

Table S5. Baseline characteristics after weighting

	No-Prescription (*n* = 65,446)	Short group (*n* = 64,085)	Long group (*n* = 64,043)	*P*-value
Age (years)	60.7 ± 0.1	60.6 ± 0.1	60.8 ± 0.1	0.566
Male sex	39,465 (60.3%)	38,019 (59.3%)	37,980 (59.3%)	0.133
Body mass index (kg/m^2)	22.7 ± 0.0	22.7 ± 0.0	22.7 ± 0.0	0.258
Hemodialysis vintage (months)	52 ± 0	52 ± 1	52 ± 0	0.981
Diabetes as underlying cause of ESKD	30,184 (46.1%)	28,964 (45.2%)	29,076 (45.4%)	0.238
CCI score	7.4 ± 0.0	7.5 ± 0.0	7.5 ± 0.0	0.012
Arteriovenous fistula	55,883 (85.4%)	54,443 (85.0%)	54,661 (85.4%)	0.476
Kt/V_{urea}	1.52 ± 0.00	1.52 ± 0.00	1.52 ± 0.00	0.795
Ultrafiltration volume (L/session)	2.30 ± 0.00	2.29 ± 0.01	2.29 ± 0.01	0.697
Hemoglobin (g/dL)	10.7 ± 0.0	10.7 ± 0.0	10.7 ± 0.0	0.698
Serum albumin (g/dL)	4.0 ± 0.0	4.0 ± 0.0	4.0 ± 0.0	0.425
Serum phosphorus (mg/dL)	4.97 ± 0.01	4.97 ± 0.01	4.94 ± 0.01	0.737
Serum calcium (mg/dL)	8.87 ± 0.00	8.87 ± 0.01	8.86 ± 0.01	0.488
Serum creatinine (mg/dL)	9.45 ± 0.01	9.44 ± 0.03	9.46 ± 0.02	0.767
RASB use	42,567 (65.0%)	42,508 (66.3%)	42,556 (66.4%)	0.023
Aspirin use	16,821 (25.7%)	16,718 (26.1%)	17,082 (26.7%)	0.146
Clopidogrel use	9,759 (14.9%)	9,553 (14.9%)	10,145 (15.8%)	0.031
Statin use	29,864 (45.6%)	29,934 (46.7%)	30,130 (47.0%)	0.041
H_2 receptor-blocker use	22,060 (33.7%)	22,169 (34.6%)	22,487 (35.1%)	0.034
MI or CHF	30,744 (47.0%)	29,748 (46.4%)	30,263 (47.3%)	0.333

Data are presented as means ± standard errors for continuous variables and numbers (percentages) for categorical variables. *P*–values were calculated using a general linear model with a complex survey design that included sample weights.

Abbreviations: CCI, Charlson Comorbidity Index; CHF, congestive heart failure; ESKD, end-stage kidney disease; MI, myocardial infarction; RASB, renin–angiotensin system blocker; No-Prescription, patients without a proton pump inhibitor prescription within 1 year; Short, patients with a prescription for < 60 days within 1 year; Long, patients with a prescription for ≥ 60 days within 1 year.

Table S6. Proton pump inhibitor prescription and any cancer or mortality in the weighted cohort.

Outcome and exposure	Univariable		Multivariable	
	HR (95% CI)	*P*-value	HR (95% CI)	*P*-value
**Any-cancer**				
Ref: No-Prescription				
Short	1.08 (1.04–1.11)	<0.001	1.07 (1.04–1.11)	<0.001
Long	1.09 (1.05–1.12)	<0.001	1.08 (1.05–1.12)	<0.001
Ref: Short				
Long	1.01 (0.98–1.04)	0.528	1.01 (0.98–1.04)	0.576
**Mortality**				
Ref: No-Prescription				
Short	0.93 (0.89–0.97)	0.001	0.95 (0.91–0.99)	0.034
Long	0.87 (0.83–0.91)	<0.001	0.90 (0.86–0.94)	<0.001
Ref: Short				
Long	0.93 (0.89–0.98)	0.002	0.95 (0.91–0.99)	0.018

Multivariable models were adjusted for age, sex, vascular access type, hemodialysis vintage, underlying cause of end-stage kidney disease, Charlson Comorbidity Index score, Kt/V_{urea}, ultrafiltration volume, hemoglobin, and serum albumin, creatinine, phosphorus, and calcium; use of a renin–angiotensin system blocker, statins, clopidogrel, aspirin, or H_2-receptor blockers; and presence of myocardial infarction or congestive heart failure. Models were fitted using the enter method.

Abbreviations: HR, hazard ratio; CI, confidence interval; No-Prescription, patients without a proton pump inhibitor prescription within 1 year; Short, patients with a prescription for < 60 days within 1 year; Long, patients with a prescription for ≥ 60 days within 1 year.

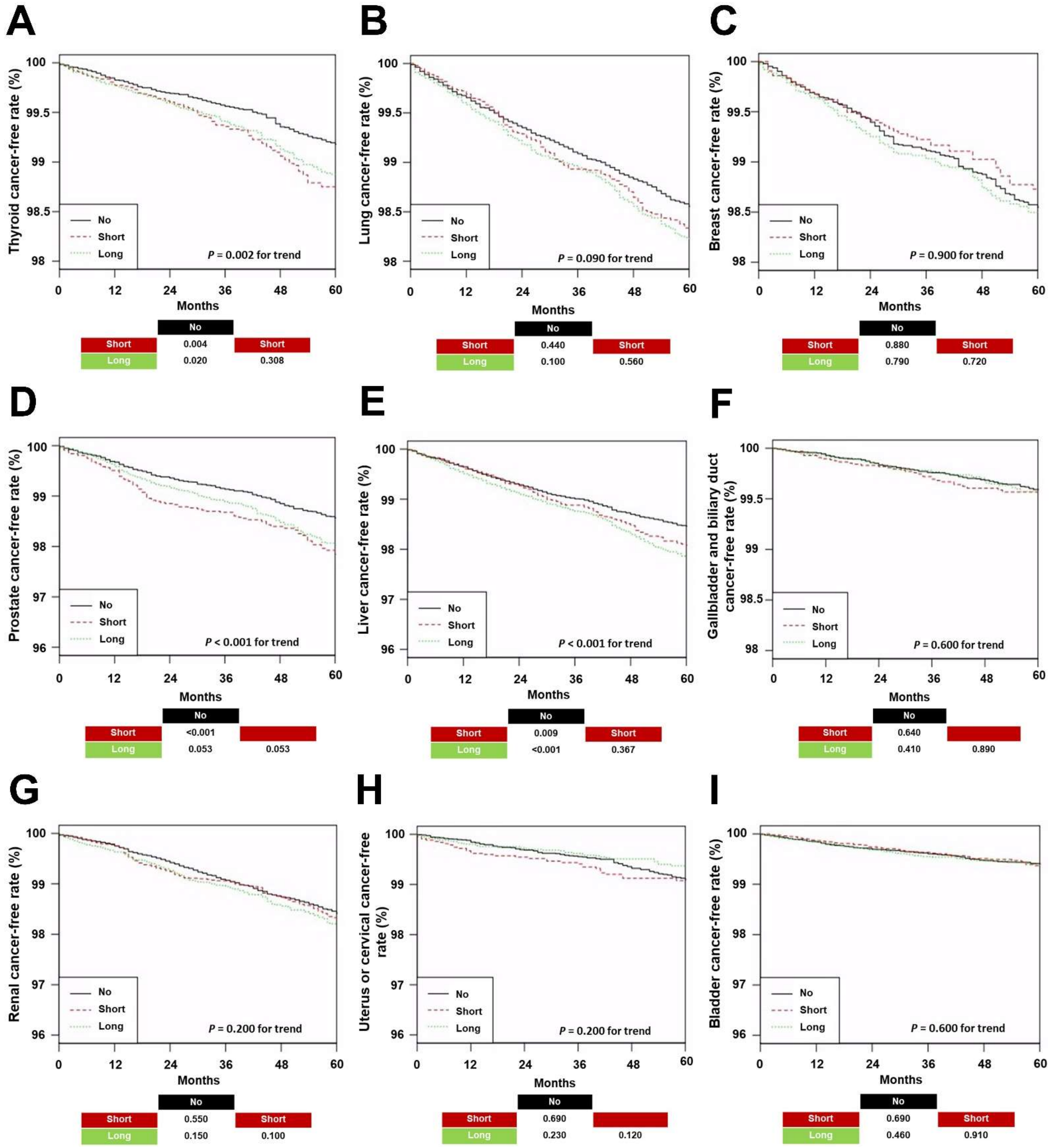

Figure S1. Kaplan–Meier curves of cancer-free rates by proton pump inhibitor use. Cancer-free rates for (A) thyroid, (B) lung, (C) breast, (D) prostate, (E) liver, (F) gallbladder and biliary duct, (G) renal, (H) uterine or cervical, and (I) bladder cancer.

P-values for pairwise comparisons with log-rank tests were presented at the bottom of the graph.

Abbreviations: No, patients without proton pump inhibitor prescription for 1 year; Short, patients with prescription for < 60 days in 1 year; Long, patients with prescription for ≥ 60 days in 1 year.

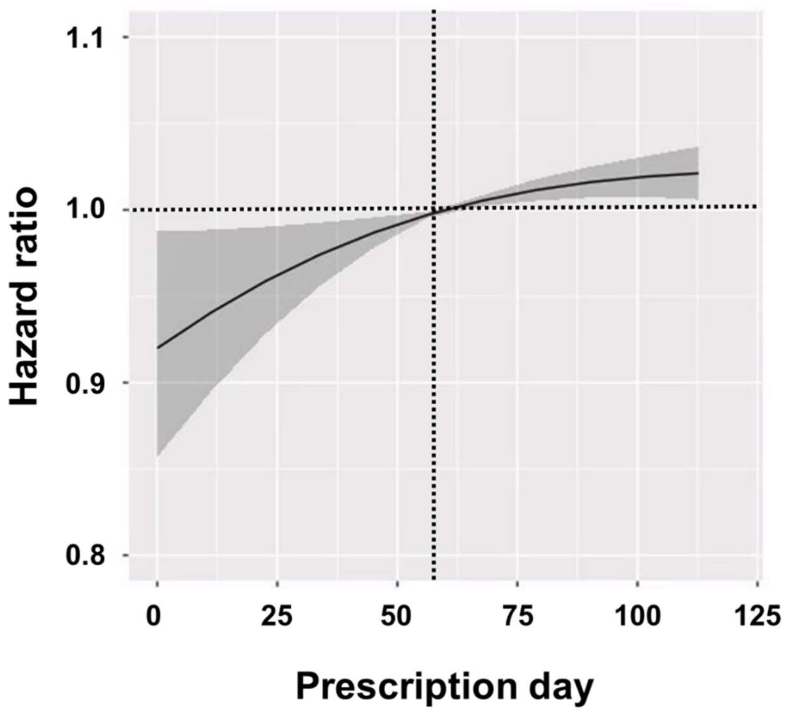

Figure S2. Spline curve plotting hazard ratio and 95% confidence interval for cancer incidence according to prescription days of proton pump inhibitors. The reference point was established at 60 prescription days.

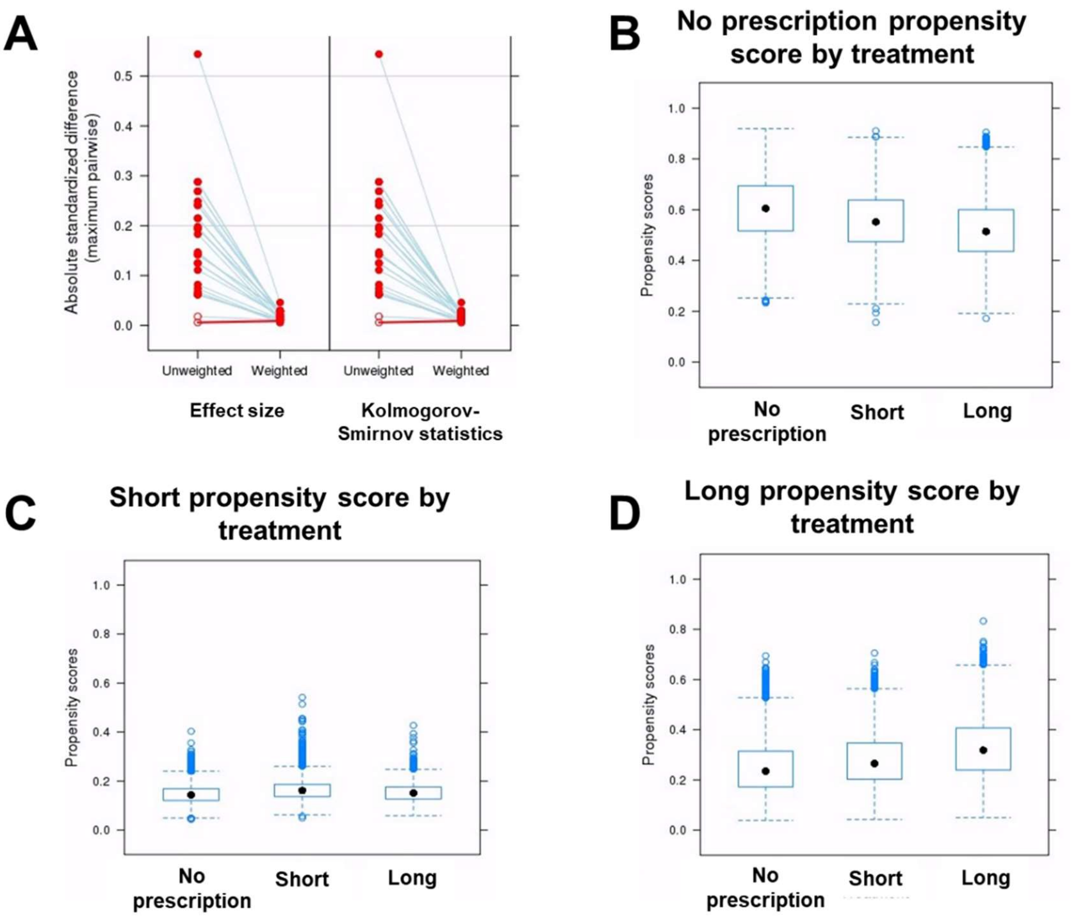

Figure S3. Balance test. (A) Absolute standardized difference plots for estimating propensity scores used to generate weights. (B-D) Boxplots illustrating the spread of propensity scores by treatment group. The filled black circles indicate the median propensity score in each treatment group. As shown, propensity score distributions overlapped substantially.

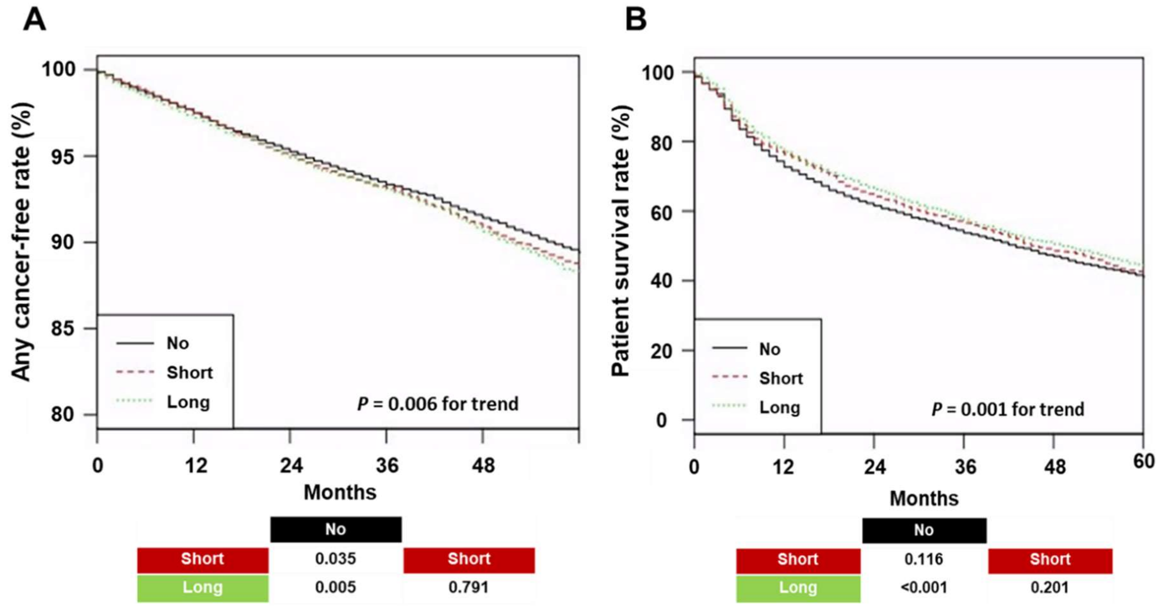

Figure S4. Kaplan–Meier curves for any cancer-free survival and overall survival by proton pump inhibitor use. (A) Any cancer-free survival in the weighted cohort; (B) overall survival among patients with cancer. Pairwise log-rank *P*-values are shown at the bottom of the graph. The cancer-free rates were 89.4%, 88.7%, and 88.2% in the No-Prescription, Short, and Long groups, respectively (Figure A). The survival rates among patients with cancer were 41.2%, 42.2%, and 44.3% in the No-Prescription, Short, and Long groups, respectively (Figure B).

Abbreviations: No, patients without a prescription for a proton pump inhibitor use in a given year; Short, patients with prescriptions for < 60 days in a given year; Long, patients with prescriptions for ≥ 60 days in a given year.
